# Oncolytic adenoviruses coated with MHC-I tumor epitopes for a new oncolytic vaccine platform

**DOI:** 10.1186/2051-1426-3-S2-P333

**Published:** 2015-11-04

**Authors:** Cristian Capasso, Mari Hirvinen, Mariangela Garofalo, Dmitrii Romaniuk, Lukasz Kuryk, Teea Sarvela, Andrea Vitale, Maxim Antopolsky, Aniket Magarkar, Tapani Viitala, Teemu Suutari, Alex Bunker, Marjo Yliperttula, Arto Urtti, Vincenzo Cerullo

**Affiliations:** 1 University of Helsinki, Helsinki, Finland; 2 University of Naples Parthenope, Naples, Italy

## Background

Oncolytic adenoviruses (OAds) demonstrated great potential for the treatment of cancer in many pre-clinical studies. The pre-existing immunity and their immunodominance, however, still limit the success of oncolytic virotherapy in cancer patients. Thus, a transition from oncolytic agents to oncolytic vaccines seems to be necessary, since the involment of the immune system may play a key role in the efficacy of OAds.

## Methods

Therefore, we developed a new oncolytic vaccine platform that uses the virus as adjuvant for MHC-I tumor epitopes that are loaded onto the viral capsid via electrostatic interactions. By using the model epitope SIINFEKL, we studied the efficacy of the peptide-coated conditionally replicating adenoviruses (PeptiCRAds) in immunocompetent mice bearing B16-OVA melanomas.

## Results

**Figure 1:**
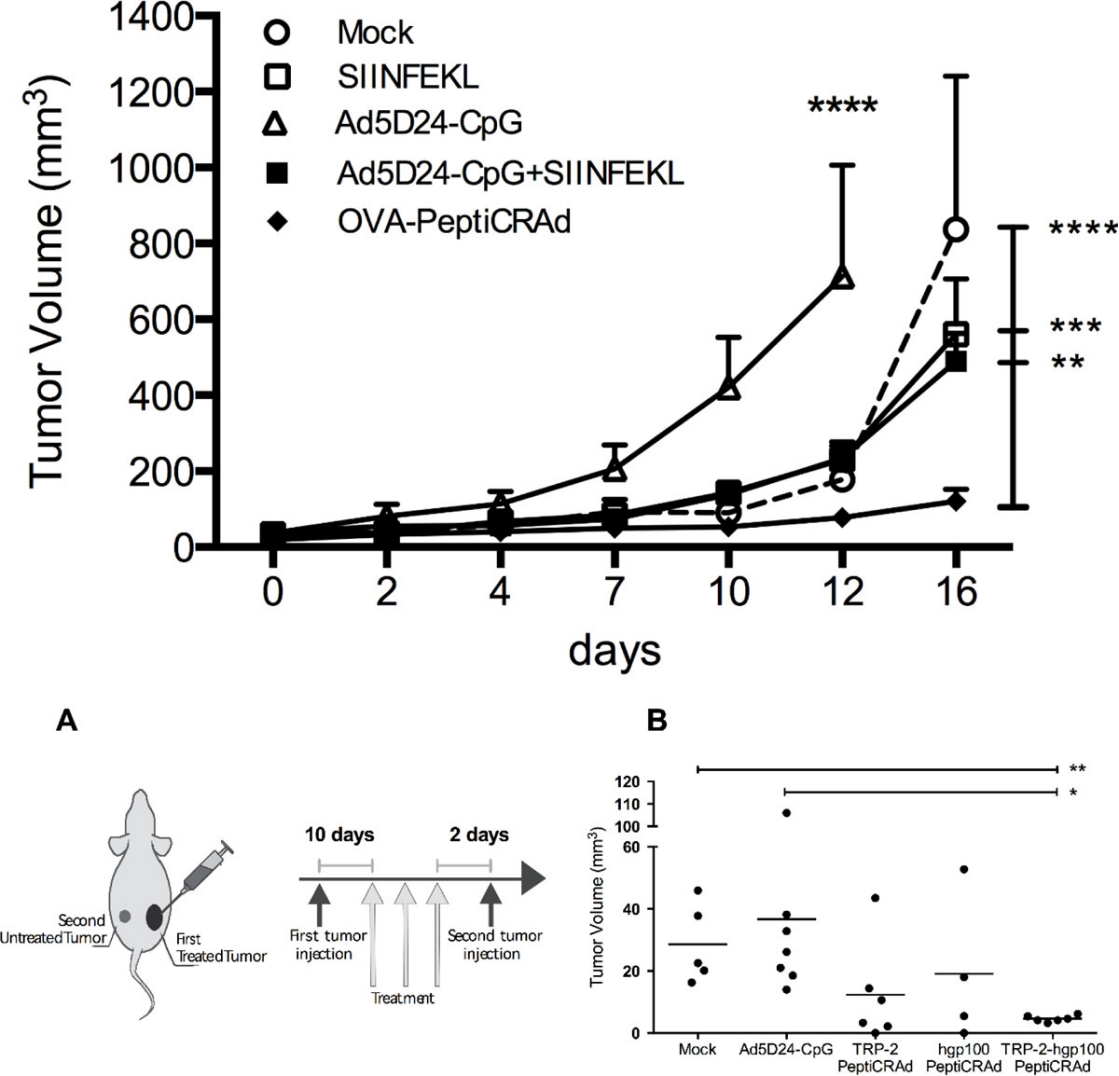


We found that OVA-targeting PeptiCRAds significantly reduced the tumor growth compared to controls (Figure 1) and elicited larger SIINFEKL-specific CD8 responses. Furthermore, we observed an increased presence of mature and epitope-specific dendritic cells in the spleens of mice treated with PeptiCRAd suggesting a modulating effect on antigen presenting cells. Next, we demonstrated that a PeptiCRAd targeting both TRP-2 and gp100 tumor antigens could elicit a broad immunological response able to significantly reduce the tumor growth of treated and, most importantly, untreated B16-F10 melanomas (Figure 2 A, B). Finally, for the first time, immune deficient mice bearing human melanomas and engrafted with a human immune system were used to study the synergy between oncolytic activity and immunogenicity of oncolytic adenoviruses. A MAGE-A1-targeting PeptiCRAd, encoding for granulocyte-macrophage colony stimulating factor, was able to eradicate human melanomas and increased the percentage of human MAGE-A1-CD8 T cells compared to the naked OAd.

## Conclusions

In conclusion, we demonstrated that the immunogenicity of OAds can be exploited to drive the immune response towards choosen epitopes and that this approach allows for rapid re-targeting without the need of genetic modifications, making PeptiCRAds a suitable platform for the next generation of highly personalized cancer vaccine immunotherapies.

